# Contrasting Effects of Sleep Restriction, Total Sleep Deprivation, and Sleep Timing on Positive and Negative Affect

**DOI:** 10.3389/fnbeh.2022.911994

**Published:** 2022-08-17

**Authors:** John A. Groeger, June C-Y. Lo, Nayantara Santhi, Alpar S. Lazar, Derk-Jan Dijk

**Affiliations:** ^1^Department of Psychology, Nottingham Trent University, Nottingham, United Kingdom; ^2^Centre for Sleep and Cognition, Yong Loo Lin School of Medicine, National University of Singapore, Singapore, Singapore; ^3^Northumbria Sleep Research Laboratory, Department of Psychology, Northumbria University, Newcastle upon Tyne, United Kingdom; ^4^School of Health Sciences, University of East Anglia, Norwich, United Kingdom; ^5^Surrey Sleep Research Center, University of Surrey, Guildford, United Kingdom; ^6^UK Dementia Research Institute, Care Research and Technology Center, Imperial College London and University of Surrey, Guildford, United Kingdom

**Keywords:** mood, emotion, sleep restriction, sleep deprivation, circadian rhythm, forced desynchrony, affect

## Abstract

Laboratory-based sleep manipulations show asymmetries between positive and negative affect, but say little about how more specific moods might change. We report extensive analyzes of items from the Positive and Negative Affect Scale (PANAS) during days following nights of chronic sleep restriction (6 h sleep opportunity), during 40 h of acute sleep deprivation under constant routine conditions, and during a week-long forced desynchrony protocol in which participants lived on a 28-h day. Living in the laboratory resulted in medium effects sizes on all positive moods (Attentiveness, General Positive Affect, Joviality, Assuredness), with a general deterioration as the days wore on. These effects were not found with negative moods. Sleep restriction reduced some positive moods, particularly Attentiveness (also General Positive), and increased Hostility. A burden of chronic sleep loss also led to lower positive moods when participants confronted the acute sleep loss challenge, and all positive moods, as well as Fearfulness, General Negative Affect and Hostility were affected. Sleeping at atypical circadian phases resulted in mood changes: all positive moods reduced, Hostility and General Negative Affect increased. Deteriorations increased the further participants slept from their typical nocturnal sleep. In most cases the changes induced by chronic or acute sleep loss or mistimed sleep waxed or waned across the waking day, with linear or various non-linear trends best fitting these time-awake-based changes. While extended laboratory stays do not emulate the fluctuating emotional demands of everyday living, these findings demonstrate that even in controlled settings mood changes systematically as sleep is shortened or mistimed.

## Introduction

The contribution of sleep and circadian rhythmicity to “mood” and cognition has been documented in many studies in healthy participants and people living with mood and cognitive disturbances (Musiek and Holtzman, 2016; Hertenstein et al., 2019). This summary of many important findings conceals a range of conceptual and methodological shortcomings. Our intention here is to clarify what we know about sleep loss and emotion in the laboratory, as well as identifying some limitations of the approaches typically used when investigating these relationships. Specifically, through extensive re-analysis of several datasets, we aim to quantify differences in feelings (Joviality, Self-assurance, Attentiveness, Fear, Hostility and Guilt, as well as less specific aspects of General Positive and Negative Affect), assessed at various times of day during standard sleep restriction, total sleep deprivation and forced desynchrony protocols. Before considering these in detail, we address some of the conventions which influence the design and conduct of studies in this area.

For very sound reasons, our studies of the effects of sleep restriction, total sleep deprivation or mistimed sleep (i.e., circadian effects), on cognition and “emotion” require repeated measurement throughout protocols. This inevitably constrains the tasks which can be used, and as a consequence, the conclusions that can be drawn about the effects of reducing, removing, or re-scheduling sleep on waking functioning are limited to tasks with particular characteristics (i.e., brief, have no or known learning effects, maintain participant motivation, avoid task related fatigue, or any of a variety of other confounds). These necessary methodological constraints may have undesirable theoretical consequences, which is a particular issue for the measurement of states which are themselves, by definition, transient. Furthermore, few studies have used identical tools across protocols designed to assess effects of repeated sleep restriction, acute total sleep loss and desynchrony between circadian rhythmicity and sleep-wake timing.

As may be clear from the previous paragraph, the terms “affect,” “mood,” and even “emotion,” are sometimes used interchangeably. This does little to establish conceptual clarity (Barrett and Russell, 1999; Kaufmann et al., 2020). DSM-V (American Psychiatric Association, 2013) defines “affect” as “a pattern of observable behaviors that is the expression of a subjectively experienced feeling state (emotion),” examples of which include “sadness, elation, and anger. (p.817).” Affect is contrasted with “mood,” which is a “pervasive and sustained emotion that colors the perception of the world. Common examples of mood include depression, elation, anger, and anxiety” (p.824). Mood and affect are also distinguished in terms the time course over which they both typically change from “seconds and minutes” in the case of affect, to days, weeks, or months, in the case of mood (see Kaufmann et al., 2020). Of course, affect and mood will both be readily impacted by life circumstances, but typically our constant routines are designed to avoid such challenges, and thus we are more likely to observe changes in affect, rather than mood in our laboratory studies.

It is also important to recognize that conceptual space described by the terms affect, emotion and mood has long been considered to be a combination of bi-polar dimensions. Beginning with Wundt (1912/1924), one of these dimensions has tended to reflect a feeling of, or lack of, activation or energy, and almost evert theorist since includes an “arousal” component in their construal of mood. Gold-standard measures of subjective sleepiness (Akerstedt et al., 2013), show that sleepiness increases with time awake, and is also strongly modulated by circadian phase. Given this, the ‘energetic’ aspect of affect would be expected to change systematically when sleep is restricted, completely lost, or permitted at different circadian phases. Whether this is a change in mood, *per se*, or a change in perceived alertness/sleepiness, is obviously problematical, and the difficulty of interpreting results is compounded where only a single measure of whatever is currently-felt this “mood” is available.

There is greater diversity in how the second general dimension of affect has been understood. For Russell (1980) the bi-polar dimensions are Arousal-Sleep and Misery-Pleasure; for Larsen and Diener (1992) they are High Activation-Low Activation and Unpleasant-Pleasant, while for Thayer (1989) these reflect Energy-Tiredness and Tension-Calmness. The first dimension in each case has clear conceptual overlap with sleep loss and reduced arousal. The second dimensions are not obviously related to these, except, perhaps Tension-Calmness. A fourth influential approach, as represented by PANAS, also invokes a bi-polar structure with poles based on Positive Affect, a combination of Pleasantness and High Activation, and Negative Affect, a combination of Unpleasantness and High Activation. That is, both dimensions are in principle affected by activation-level. Despite this, Watson and Tellegen (1985), make explicit that these two dimensions are orthogonal, which is more implicit in the other frameworks. These accounts suggest alternative predictions. If the two dimensions are truly orthogonal, it is unclear why the dimension construed as Misery-Pleasure, Unpleasant-Pleasant or Tension-Calmness should change when people are under-slept or awake when they would typically be asleep. In contrast, because activation is intrinsic to both Positive and Negative Affect in the Watson and Tellegan approach, both dimensions of affect might be expected to change.

In our previous studies, data from some of which are re-analyzed below, we have shown that acoustic suppression of Slow Wave Activity results increased daytime sleepiness and reduced Positive Affect (Dijk et al., 2010; Groeger et al., 2014), as does sleep restriction and sleep deprivation (Lo et al., 2012), and rescheduling sleep and wakefulness to take place at atypical circadian phases (Santhi et al., 2016). None of these manipulations resulted in a change in Negative Affect. These findings provide an important confirmation of the orthogonality of Positive and Negative Affect, given that one of the dimensions changes while the other does not. However, these findings are also problematical for the PANAS framework, since lowered energy levels might be expected to influence both Positive and Negative Affect, rather than just one dimension. However, it is also possible that the measurement of Negative Affect is simply insensitive to changes brought about by sleep loss. Other studies, described below, using alternative measurement techniques report increases in what might be regarded as negative mood as a function of sleep loss. For this reason, the sensitivity, or otherwise, of negative affect to sleep manipulations is a particular focus of this paper.

Studies have typically adopted one of two broad measures of affective state, such as PANAS or POMS^1^, or used bespoke single item rating scales. Although the latter are convenient, inter- and intra-individual differences in how different words are construed, and the lack of information about how each construal might relate to other states, make findings difficult to interpret within larger theoretical frameworks.

Perhaps because of their length, there is a dearth of information regarding the effects of accumulating sleep loss on more specific aspects of mood using measures which have the scope to elucidate more nuanced changes in mood such as the full versions of PANAS or POMS. There are exceptions, certainly, such as Meney et al. (1988) who showed that the effect of one night of sleep loss increased Confusion and Fatigue and reduced Vigor, i.e., there were effects of sleep loss on both negative and positive affect. Similar findings were reported by Dinges et al. (1997), as well as increased Tension, when sleep was restricted to 5 h per night for one week. Notably these results were consistent across Morning, Afternoon or Evening testing- implying that circadian influences on mood are weak or absent when sleep is restricted, although no assessment were obtained during the nighttime. Consistent with this there were no time-of-day effects in a similar 5-h restriction study reported recently by Harous et al. (2021), but in their case only Fatigue-Inertia, but no other aspects of negative mood increased. Other studies, also using PANAS (e.g., Saksvik-Lehouillier et al., 2020) show no effect of sleep restriction (2 h less than normal sleep duration) on Negative Affect, but, as in our own work, a reduction in Positive Affect.

There are fewer studies which use either of the major pan-mood measures when investigating the effects of total sleep loss. Li et al. (2021) is an important recent exception. After 36 h awake, between 08:00 and late evening the following day, POMS measured mood showed significant deterioration in mood. Specifically, anxiety, anger, fatigue and confusion increased, although depression did not change, whereas vitality decreased significantly. Moreover, fMRI data collected by Li and colleagues show that changes observed in subjective mood were reflected in changes in thalamic and inferior frontal activity-brain areas which are consistently implicated after acute sleep loss (e.g., Vandewalle et al., 2009). Similarly, an earlier acute sleep deprivation study by Kaida and Niki (2014), also shows an increase in negative affect (POMS: Sleepiness, Confusion, Fatigue, and Anger) and a decrease in positive affect (i.e., Vitality), when mood was assessed at 8AM and 10PM the following day. Unfortunately, a 36-h delay between the collection of mood data inevitably confounds what might be independent and interacting effects of extended wakefulness and circadian phase.

Time of day effects are also problematical, for similar reasons, in multi-day studies of mood and sleep conducted as people live their everyday lives. Thus, for example, Shen et al. (2022) report an impressive 28-day long study of adolescent sleep and mood, but while subjective sleep quality was assessed on waking, a shortened and adapted version of PANAS-X was administered only in the afternoon/early evening. While mood was assessed more frequently by Wong et al. (2021), the intriguing day-to-day bi-directional effects of mood and sleep across consecutive days cannot easily distinguish between what may be cumulative or compensatory circadian and homeostatic influences.

The examples of empirical studies cited above illustrate three consistent shortcomings of experimental studies of the relationship between sleep and mood. Firstly, within POMS and in studies using single item scales, positive mood is synonymous with energy, arousal, or activation, but is measured only with a single scale. In addition to being confounded with feelings of sleepiness or diminished alertness, a different measurement approach, such as that offered by PANAS, is required if the effects on more nuanced aspects of positive affect are to be explored. Secondly, there are inconsistencies in relation to which aspects of negative mood are affected by sleep loss. Finally, level of activation is intrinsic to understandings of mood, but it is also quintessentially circadian. Most studies of the mood-sleep relationship, if not all, confound time awake, time of day and circadian phase, each of which are known to affect subjective alertness.

The data reanalyzed below come from two separate studies which were carried out in order to assess the effects of sleep restriction, sleep deprivation (Lo et al., 2012) and misalignment of the sleep-wake cycle with the circadian system (Santhi et al., 2016), on repeated performance of tests of cognitive and affective functioning, and how any effects were modulated by a polymorphism of the Period3 gene. Overall these studies showed that measures of alertness and sustained attention were very sensitive to the sleep manipulations whereas tests which were more demanding on executive resources were not very sensitive to these manipulations, but were sensitive to the effects of the polymorphism (Groeger et al., 2008). With regard to measures of affect it was notable and that while Positive Affect, as measured with PANAS, reduced as sleep pressure increased, Negative Affect was at a low level and appeared impervious to the manipulations carried out. No attempt was made in those analyzes to decompose these broad measures of affect into more discrete components, and this paper seeks to redress this. We trust that comparative data from sleep restriction, total sleep deprivation and forced desynchrony protocols provide a unique insight into how different aspects of Positive and Negative Affect vary across waking states.

## Materials and Methods

Full methodological details are provided in the original reports of both studies, some of the more relevant details are rehearsed here for the reader’s convenience.

### Procedures

**FIGURE 1:**
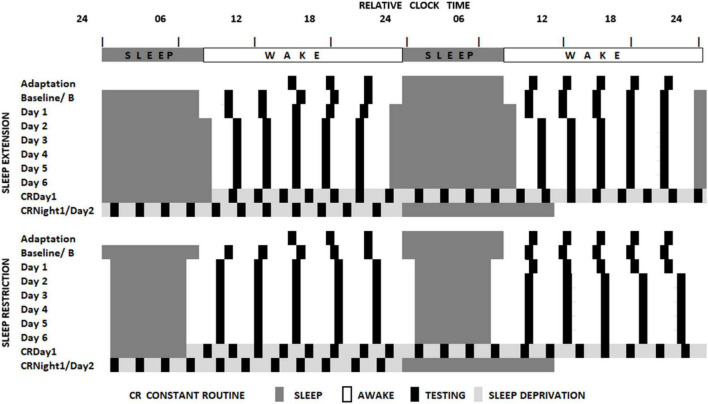
Schematic representation of the sleep restriction-sleep extension protocol. All participants completed two sleep opportunity condition sessions. In both sessions, participants were scheduled to sleep at their habitual times for two consecutive nights and then to extended (10 h) or restricted (6 h) sleep opportunities for 7 consecutive days. This then was followed by a sleep deprivation under constant routine conditions (see Duffy and Dijk, 2002) and a recovery sleep opportunity. Black boxes indicate the timing of the cognitive assessments (Lo et al., 2012).

**FIGURE 2:**
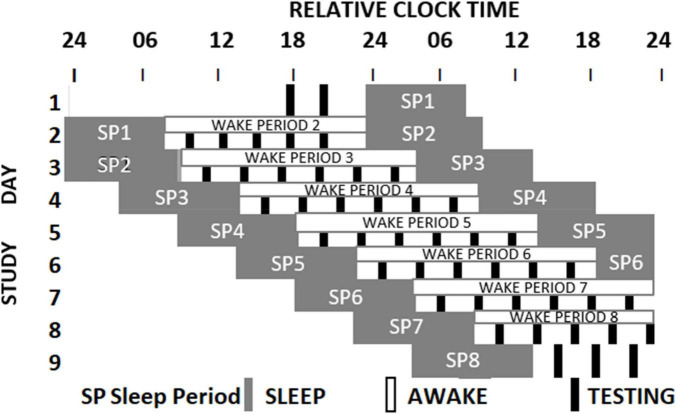
Schematic representation of the Forced Desynchrony protocol. After two baseline days participants were scheduled to a 28-h sleep wake cycle by extending the sleep opportunity to 9h:20 min and the wake period to 18h:40 min. Light intensity was low during wake periods. During each wake period 6 assessments of cognition and mood (black boxes) were conducted. The melatonin rhythm (not shown) cannot follow the 28-h sleep-wake cycle and oscillates at it near 24-h intrinsic period. As a consequence, mood assessments occur at different circadian times (Santhi et al., 2016).

The sleep loss study (see Figure 1 and Lo et al., 2012) required that participants visited the laboratory on two extended occasions at least 10 days apart. On the first or second occasion, depending on counterbalancing, after habituation and baseline nights (8h time in bed), participants were assigned to a seven-night regime allowing 6 or 10 h of sleep opportunity per night, followed by a 41- or 39-h period of extended waking, and a recovery night where 12 h of sleep was permitted. Over the restriction/extension week, an extensive battery of cognitive tests, including PANAS, was undertaken on five equally spaced occasions between sleeps; and every 2 h during sleep deprivation. The forced-desynchrony study (see Figure 2 and Santhi et al., 2016) required a single extended visit to the laboratory, which, after normal days and nights (8 h in bed scheduled to participant’s typical bed-time), required that participants experienced a 9 h:20 min sleep opportunity followed by a period of continuous wakefulness of 18 h:40 min, for seven consecutive cycles, meaning that participants would begin by sleeping and waking at a typical day/night time, and then sleep and wake progressively later until sleeping and waking once again at the original times. The same test battery was administered at approximately every 3 h after waking. Throughout both protocols all participants lived in light-controlled environments. During the forced desynchrony study and during the sleep-deprivation/constant routine segment of the sleep restriction/extension study light levels were low (i.e., lux < 10). During the other days of the sleep-extension/sleep restriction study participants were exposed to normal indoor ambient light.

### Participants

Thirty-six healthy individuals (18 males; Mean age = 27.6, SD = 4.0 years) completed the sleep loss study protocol. A different group of 34 participants (34; 18 women; Mean age = 25.54, SD = 3.323 years) completed the forced-desynchrony protocol.

### Measurement of Mood and Statistical Analyzes

The original 20-adjective version of PANAS was administered as part of the computerized test battery. Ten of these adjectives assess Positive and ten Negative Affect. However, these adjectives are all components of the more specific mood measurement possible with the much longer PANAS-X. The reanalysis reported below assigned the PANAS adjectives to the PANAS-X mood classifications (see Table 1), and then averaged responses to provide a single measure for each classification. The Cronbach alpha for each of these new components were calculated for each of these new scales, by timepoints across individuals, showing excellent reliabilities in each dataset. This allows us to assess change in three positive (Joviality, Self-Assurance, Attentiveness) and negative (Fear, Hostility, Guilt) moods reported upon below, as well as the for the now, far briefer, measures of General Negative and Positive Affect (see Table 1).

**TABLE 1:** Adjectives contributing to measurement of specific Negative and Positive moods and their reliabilities.

Valence	Mood	Adjectives included in 20-item PANAS	Cronbach’s Alpha Reliabilities	Adjectives comprising PANAS-X
Negative	General Negative Affect	upset, distressed	SE:.98; SR:.97 SD:.99; FD:.99	*Included in negative moods (Fear, Hostility, Guilt)*
	Fear	afraid, scared, nervous, jittery	SE:.98; SR:.96 SD:.98; FD:.98	frightened, shaky
	Hostility	hostile, irritable	SE:.97; SR:.97 SD:.97; FD:.98	angry, scornful, disgusted, loathing
	Guilt	guilty, ashamed	SE:.97; SR:.90 SD:.96; FD:.99	blameworthy, angry at self, disgusted with self, dissatisfied with self
Positive	General Positive Affect	active, inspired, interested	SE:.99; SR:.99 SD:.99; FD:.98	*Included in positive moods (Joviality, Self-Assurance, Attentive)*
	Joviality	excited, enthusiastic	SE:.99; SR:.99 SD:.99; FD:.96	happy, joyful, delighted, cheerful, lively, energetic
	Self-Assurance	proud, strong	SE:.99; SR:.99 SD:.99; FD:.99	confident, bold, daring, fear
	Attentive	alert, attentive, determined	SE: .97; SR: .98 SD:.98; FD: .97	Concentrating

*NB: Nota bene; SR/E: Sleep Restriction/Extension; SD: Sleep Deprivation; FD: Forced Desynchrony.*

Repeated Measures ANOVA were carried out using SPSS v.28 (IBM Corp, 2021). For each of the eight moods defined in Table 1, separate analyzes contrasted mood changes as a function of (a) Sleep Restriction/Sleep Extension, with 5 time of day test-points for each of 8 days; (b) Total Sleep Deprivation following Sleep Extension or Sleep Restriction, with 18 or 19 time points reflecting the two-hourly test batteries across the sleep deprivation, and eight 28-h Forced Desynchrony “days”, each with six equally spaced test battery administrations.

There were small amounts of missing PANAS data in each protocol (Sleep Restriction/Extension, 2.5%; Sleep Deprivation: 2.7%; Forced Desynchrony: 3.8%), which Missing Value Analyzes showed to be random. Multiple imputation based on linear regression was used to ensure participants missing minimal data could be included in the analyzes. Main effects and interactions were decomposed to simple effects and followed up with Bonferroni α-adjusted *post hoc* contrasts as appropriate. Time of day and Forced Desynchrony day simple effects were further assessed using linear or more complex contrasts in order to assess which shaped trend best represented any changes across the protocol. Effect sizes were quantified using partial-eta-squared (η*_*p*_^2^*, where 0.01 indicates a small effect; 0.06 indicates a medium effect and 0.14 indicates a large effect, Cohen, 1988).

## Results

The effects of sleep restriction, sleep deprivation and forced desynchrony of wake and sleep times on different aspects of positive and negative affect are considered in turn.

### Sleep Restriction

**FIGURE 3:**
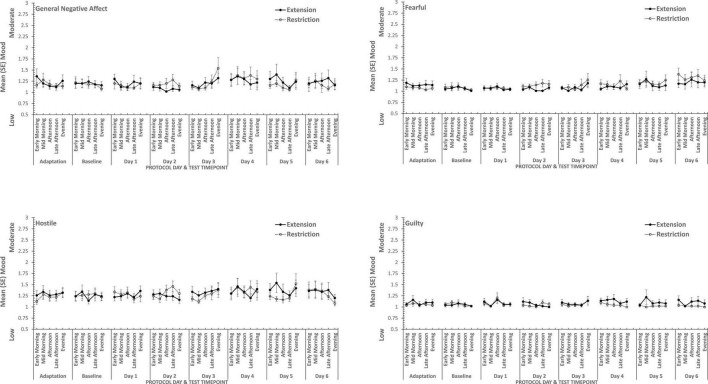
Effects of sleep restriction and extension on Negative Mood (General Negative Affect, Fearfulness, Guilt, Hostility) across protocol days and mood assessment time points.

**FIGURE 4:**
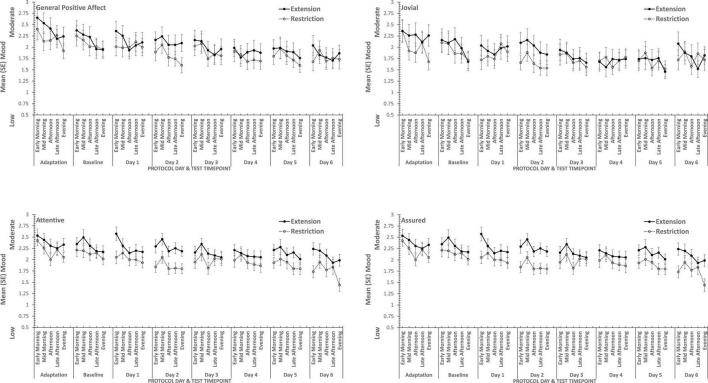
Effects of sleep restriction and extension on Positive Mood (General Positive Affect, Assuredness, Joviality, Attentiveness) across protocol days and mood assessment time points.

Overall, consistent with what was reported previously, positive moods changed considerably across the week-long sleep restriction or extension (see Table 2 and Figure 3), although extension or restriction *per se* had relatively little effect. However, two specific aspects of positive mood, do show main effects extension-restriction, such that participants reported being more Attentive (i.e., alert, attentive, determined) when a 10 h sleep opportunity was available, than when sleep restricted, and a small effect-size three-way interaction for General Positive Affect between day into protocol, time of day when tested and sleep opportunity condition. Negative moods, although relatively stronger or weaker than each other, show no effect of restricting sleep to 6 h per night over a week (see Table 2 and Figure 4). This is also consistent with our previous reports.

**TABLE 2:** Effects of sleep restriction and time of day on Negative and Positive moods.

Source _(df, df)_	F	η_*p*_	F	η_*p*_	F	η_*p*_	F	η_*p*_
Negative	**GNA**		**Fearful**		**Hostile**		**Guilty**	
Extension-Restriction_(1,35)_	1.292	0.035	1.288	0.035	2.696	0.07	2.224	0.058
Day_(7,245)_	1.653	0.044	1.171	0.032	1.594	0.042	1.604	0.043
Test-point_(4,140)_	0.972	0.026	1.293	0.035	0.939	0.025	0.734	0.02
Extent-Restrict * Day_(7,245)_	1.201	0.032	1.328	0.036	1.11	0.03	0.982	0.027
Extent-Restrict * Test-point_(4,140)_	1.458	0.039	1.172	0.032	0.942	0.025	0.48	0.013
Day * Test-point_(28,245)_	1.422	0.038	1.279	0.034	1.36	0.036	1.301	0.035
Extent-Restrict * Day * Test-point_(28,980)_	1.368	0.037	1.252	0.034	0.746	0.02	0.492	0.013
Positive	**GPA**		**Jovial**		**Assured**		**Attentive**	
Extension-Restriction_(1,35)_	3.746	0.094	2.127	0.056	0.663	0.018	11.388^#^	0.24
Day_(7,245)_	19.092^#^	0.347	15.028^##^	0.295	7.812^#^	0.178	13.264^##^	0.269
Test-point_(4,140)_	11.191^#^	0.237	10.724^##^	0.23	6.399^#^	0.151	12.18^##^	0.253
Extent-Restrict * Day_(7,245)_	1.119	0.03	0.953	0.026	0.774	0.021	1.759	0.047
Extent-Restrict * Test-point_(4,140)_	1.147	0.031	1.336	0.036	0.372	0.01	1.666	0.044
Day * Test-point_(28,245)_	1.402	0.037	1.908^#^	0.05	1.39	0.037	1.454	0.039
Extent-Restrict * Day * Test-point_(28,980)_	1.569*	0.042	1.177	0.032	0.849	0.023	1.272	0.034

*KEY: GNA: General Negative Affect.; GPA: General Positive Affect.*

*p<.05, **p<01, ^#^p<.005, ^##^p<.001.

η *_p_ Partial-eta squared.*

Joviality, Assuredness and General Positive Affect, all showed large, statistically significant, effects of both day-into-protocol, and time of day when tested, generally there is a steady decline in each positive mood as individuals spent more days in the laboratory, and from earlier to later in the day (see Figure 3). Only in the case of Joviality did these effects interact significantly. Within subject contrasts showed that the decline is best characterized in each case by a quadratic function for General Positive Affect (Linear: η*_*p*_^2^* = 0.24; Quadratic: η*_*p*_^2^* = 0.48; only provided where contrast is statistically significant), Attentiveness (Quadratic: η*_*p*_^2^* = 0.54), Joviality (Quadratic: η*_*p*_^2^* = 0.49) and Assuredness (Quadratic: η*_*p*_^2^* = 0.34). *Post hoc* testing for each of the positive moods showed that days 1, 2, and 3 were significantly more positive than the final three laboratory days, differing only in whether the third and fourth days themselves differed.

The four positive moods also differed significantly across the time of day when testing took place. There is a linear trend indicating a general decline across the day in positivity, which had the largest effect sizes (General Positive Affect: η*_*p*_^2^* = 0.72; Joviality: η*_*p*_^2^* = 0.62; Assuredness: η*_*p*_^2^* = 0.63; Attentiveness: η*_*p*_^2^* = 0.77). The consistency of these trends is evident in Figure 3, as is the more complex change in Assuredness, with most positivity in mid-morning, and a slight improvement in late afternoon of compared with a mid-day slump. Joviality is also subject to an interaction. Participants were more Jovial earlier in the day and less so at the end of the day. While Joviality declines across the protocol the difference between Joviality earlier and later in the day is more apparent as the protocol proceeds. For General Positive Affect, this time into protocol and time of day is also apparent, but is intensified toward the end of the protocol when sleep has been restricted. Within subject contrasts suggest that day into protocol is a quadratic, 4th Order or 6th Order trend combined with linear or quadratic effects of test-point trends (Quadratic-Quadratic: η*_*p*_^2^* = 0.16; 4th Order -Quadratic: η*_*p*_^2^* = 0.12; 6th Order-Linear: η*_*p*_^2^* = 0.15, 6th Order-Quadratic: η*_*p*_^2^* = 0.14, representing the effects of Day and Test-point, respectively).

It is important to recognize that these effects do not interact with the sleep restriction-extension manipulation, and thus reflect the constraints of being in the laboratory for an extended period or time of day, rather than the accumulation or depletion of any sleep debt or drive.

General Positive Affect is the exception to this. It also shows the general trends of time of day and day into protocol, but their combined effects are influenced by sleep restriction-sleep extension. Within subject contrasts suggest that this is best fit by a combination of linear and more complex trends (Linear-Linear-Cubic: η*_*p*_^2^* = 0.12; Linear-Linear-4th Order: η*_*p*_^2^* = 0.11; Linear-5th Order-4th Order: η*_*p*_^2^* = 0.12, representing the effects of Extension-Restriction, Day and Test-point, respectively).

In summary, restricting sleep opportunity to 6 h per night has very little effect on mood, nor does the cumulative loss of sleep across the protocol. The number of days in the laboratory, and the time of day at which mood is assessed, does influence particular positive moods, but in a very similar way for each: generally declining within the day and declining from earlier days until part way through the protocol, and then flattening. Negative moods, on the other hand, remain stable, more or less, across time of day and days in the laboratory.

### Sleep Deprivation

**FIGURE 5:**
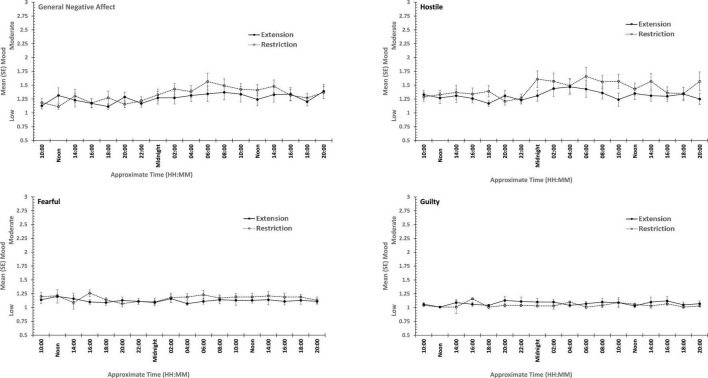
Effects of prior sleep restriction and extension on Negative Mood (General Negative Affect, Fearfulness, Guilt, Hostility) across 38 h of wakefulness.

**FIGURE 6:**
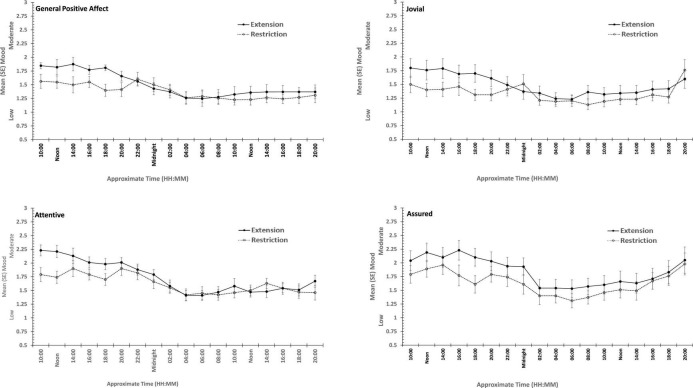
Effects of prior sleep restriction and extension on Positive Mood (General Positive Affect, Assuredness, Joviality, Attentiveness) across 38 h of wakefulness.

The same participants followed their period of sleep restriction or extension with 39/41 h continuous waking, during which PANAS was completed at fixed intervals. The data reported here are for 18 of the occasions on which they did so (matched for chronological time). Once again there are substantial effects on all four positive moods studied (see Table 3 and Figure 5), but on this occasion, General Negative Affect and Hostility also changed systematically across the protocol (see Table 3 and Figure 6).

**TABLE 3:** Effects of sleep history (extension-restriction) and accumulating sleep loss (test point during sleep deprivation) on Negative and Positive moods.

	Extension-Restriction (1,35)		Test-point (17,595)		Extension-Restriction	
					* Test-point (17,595)	
	F	η_*p*_	F	η_*p*_	F	η_*p*_
Negative						
GNA	0.433	0.015	3.118**	0.1	1.085	0.037
Fearful	1.999	0.067	0.918	0.032	0.831	0.029
Hostile	5.216**	0.157	2.386**	0.079	1.009	0.035
Guilty	0.446	0.016	0.856	0.03	1.091	0.037
Positive						
GPA	4.279*	0.133	10.018^#^	0.264	2.343**	0.077
Jovial	3.878	0.122	4.376**	0.135	1.88*	0.063
Assured	3.758	0.118	7.181^#^	0.204	0.953	0.033
Attentive	2.844	0.092	16.683^##^	0.373	4.097^##^	0.128

*KEY: GNA: General Negative Affect; GPA: General Positive Affect.*

*p<.05, **p<01, ^#^p<.005, ^##^p<.001.

η *_p_ Partial-eta squared.*

Participants were more Hostile when their sleep had been restricted the previous week. Statistically reliable effects of time awake across the protocol were present for both Hostility and General Negative Affect; both Hostility and General Negative Affect increase with time awake. Within subject contrasts of the main effect of time awake revealed that General Negative Affect had a strong linear trend, but also evidence of more complex trends (Linear: η*_*p*_^2^* = 0.23; 5th order: η*_*p*_^2^* = 0.20; 9th order: η*_*p*_^2^* = 0.17). A linear trend was also evident for Hostility, but the change in Hostility with time awake was more complex, being relatively low and flat to begin with, then Hostility increases and remains at this higher level but varies from approximately in the second half of the sleep deprivation (Linear: η*_*p*_^2^* = 0.13; Cubic: η*_*p*_^2^* = 0.18; 8th order: η*_*p*_^2^* = 0.13; 12th order: η*_*p*_^2^* = 0.14; 15th order: η*_*p*_^2^* = 0.23).

General Positive Affect was lower after a week of restricted sleep, but the effect of prior sleep loss was not statistically significant for any of the other positive moods. Each positive mood changed substantially as time awake increased (see Table 3 and Figure 5). Within subject contrasts for this main effect showed that the change in positive mood is best characterized by a linear decline for General Positive Affect (Linear: η*_*p*_^2^* = 0.53; Quadratic: η*_*p*_^2^* = 0.36; Cubic: η*_*p*_^2^* = 0.29), and Attentiveness (Linear: η*_*p*_^2^* = 0.74; Quadratic: η*_*p*_^2^* = 0.38; Cubic: η*_*p*_^2^* = 0.34), with substantially larger effect sizes for linear fits than for quadratic or more complex functions. In contrast, for Joviality (Linear: η*_*p*_^2^* = 0.14; Quadratic: η*_*p*_^2^* = 0.28; Cubic: η*_*p*_^2^* = 0.33) and Assuredness (Linear: η*_*p*_^2^* = 0.33; Quadratic: η*_*p*_^2^* = 0.43; Cubic: η*_*p*_^2^* = 0.44), non-linear trends dominate, with both Assurance and Joviality at higher levels during what would be the normal waking day, decreasing in the typical nighttime, and recovering almost to the early session levels. These main effects of time awake are subject to an interaction with prior sleep loss for General Positive Affect, Joviality and Attentiveness (see Figure 5). For General Positive Affect combination of linear and quadratic trends for sleep extension/restriction and test-point respectively has double the effect size that the only other statistically significant contrast (Linear-Linear: η*_*p*_^2^* = 0.13; Linear-Quadratic: η*_*p*_^2^* = 0.26). For each of the first five, and the final test-point, participants having had a week of sleep restriction were less positive than when they preceded the sleep deprivation with extended sleep opportunities. This pattern is similar for Joviality, except that the difference is at the penultimate rather than the final test-point, with a combination of linear trends the only statistically outcome for combination of trends (Linear-Linear: η*_*p*_^2^* = 0.21). For Assuredness the increased positivity after sleep extension occurs at the second, fourth, and fifth test-points, and is only fit by a combination of Linear and 6th order functions (Linear-6th Order: η*_*p*_^2^* = 0.11); while for Attentiveness, the statistically significant differences occur at the first, second and fifth test-points, and is again best represented by a combination of linear trends (Linear-Linear: η*_*p*_^2^* = 0.36; Linear-Quadratic: η*_*p*_^2^* = 0.18; Linear-11*^th^* Order: η*_*p*_^2^* = 0.17). The two-way interactions involving time Attentiveness and General Positive Affect are similar, in addition to the typical tendency of positive mood to decline with time awake, there is no improvement in these moods toward the end of the protocol, and mood is less positive early in the protocol when sleep has been restricted. For Joviality, unlike these other two positive moods, while the effect of prior sleep restriction is clear, so too in this case is an improvement toward the end of time awake approaches.

In summary, sleep deprivation leads to an increased negativity in mood, but only for General Negative Affect and Hostility, those who were well slept were less hostile across the sleep deprivation than when they had been sleep restricted. Positive moods were again more labile, some (Joviality and Assuredness) are more positive after sleep, become less positive as time awake increases reaching their lowest point in the morning of the next day, but then during the daytime recover to their initial levels. The same is true for General Positive Affect, but the recovery is not as complete, while Attentiveness declines steadily as time awake increases until the deterioration ceases mid-session with no recovery. These effects are exacerbated early in the session when sleep has been restricted. Thus, sleep deprivation has a far more profound effect on mood than does sleep restriction, at least at the extents studied here. However, when sleep at typical bedtimes is prevented, the accumulation of sleep loss over the previous week certainly does affect mood during extended waking. The deteriorations in at least some moods and subsequent recovery in others, raises the possibility that homeostatic sleep drive as well as circadian modulation, affect some moods more than others. The next data set allow us to establish the effects of circadian change in the near absence of sleep loss.

### Sleeping at Different Circadian Phases

Beginning at their typical sleep time, participants were scheduled to sleep 28 h later each day on seven successive occasions, with a maximum of 9.33 h of sleep permitted. Approximately every 3 h when awake, participants undertook the same battery of tests described above, including PANAS. Table 4 summarizes the outcome of repeated measures anovas on each of the eight moods considered above.

**TABLE 4:** Effects of circadian phase on Negative and Positive moods.

	Day		Test-point		Day*Test-point	
	F(6,186)	η_*p*_	F(5,198)	η_*p*_	F(30,990)	η_*p*_
Negative						
GNA	2.514*	0.073	1.11	0.034	0.908	0.028
Fearful	0.351	0.011	0.723	0.022	0.679	0.021
Hostile	2.859**	0.082	1.331	0.04	1.68**	0.05
Guilty	0.241	0.007	1.251	0.038	1.186	0.036
Positive						
GPA	10.265^##^	0.243	47.395^##^	0.597	6.001^##^	0.158
Jovial	8.698^##^	0.214	30.498^##^	0.488	4.08^#^	0.113
Assured	5.102^#^	0.138	25.06^##^	0.439	3.539^#^	0.1
Attentive	8.025^##^	0.2	54.348^##^	0.629	6.638^##^	0.172

*KEY: GNA: General Negative Affect; GPA: General Positive Affect.*

*p<.05, **p<01, ^#^p<.005, ^##^p<.001.

η *_p_ Partial-eta squared.*

**FIGURE 7:**
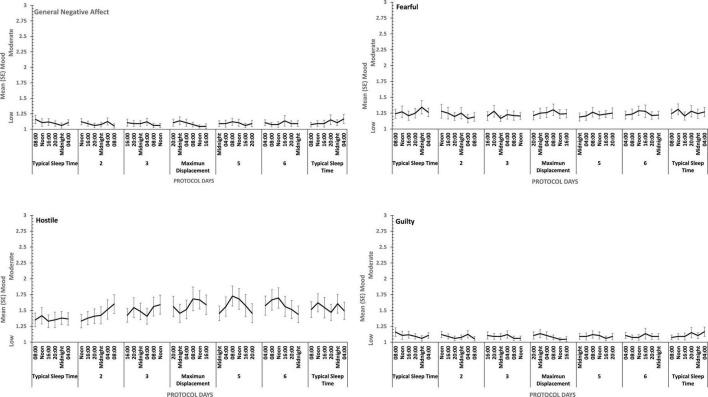
Effects of circadian phase on Negative mood (General Negative Affect, Fearfulness, Guilt, Hostility) across a week-long 28 h Forced Desynchrony protocol.

**FIGURE 8:**
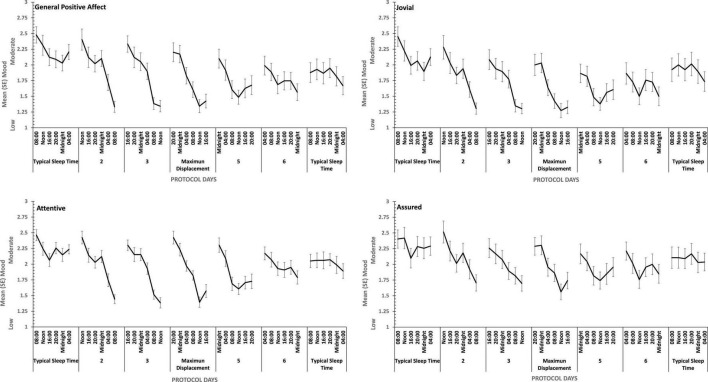
Effects of circadian phase on Positive mood (General Positive Affect, Assuredness, Joviality, Attentiveness) across a week-long 28hr Forced Desynchrony protocol.

There were main effects of study day for each of the four positive moods, and for General Negative Affect and for Hostility (see Figures 7, 8). General Negative Affect increases as individuals’ sleep and waking was displaced further from their typical timing, but neither individual comparisons of each day, nor within subject contrasts revealed significant differences or trends. Linear and quadratic contrasts for Hostility were statistically reliable for this same pattern of circadian displacement on negative affect (Linear: η*_*p*_^2^* = 0.12; Quadratic: η*_*p*_^2^* = 0.12). The point at which testing occurred in the 18.66-h period of waking in each study day also exerts a large additional influence. Figure 7 also shows that while for days at or near typical sleep-wake timing there is a flat or erratic effect of time of day, Hostility increases across the day (days 2, 3, 4), and decreases across the later part of the day (days 5 and 6). This occurs as maximum displacement from typical sleep time approaches and recedes.

Strong quadratic trends are evident in the main effects of circadian displacement on General Positive Affect (Linear: η*_*p*_^2^* = 0.24; Quadratic: η*_*p*_^2^* = 0.40), Joviality (Linear: η*_*p*_^2^* = 0.10 Quadratic: η*_*p*_^2^* = 0.49), Assuredness (Linear: η*_*p*_^2^* = 0.10; Quadratic: η*_*p*_^2^* = 0.32) and Attentiveness (Linear: η*_*p*_^2^* = 0.10; Quadratic: η*_*p*_^2^* = 0.54), and no more complex trends were statistically reliable. Positive mood reduces the further from typical sleep timing the observations on a given study are made. In contrast, linear trends best represent the effect of test-point for each positive mood (General Positive Affect, Linear: η*_*p*_^2^* = 0.72; 5th Order: η*_*p*_^2^* = 0.17; Jovial, Linear: η*_*p*_^2^* = 0.62; 5th Order: η*_*p*_^2^* = 0.29; Assuredness, Linear: η*_*p*_^2^* = 0.63; 5th Order: η*_*p*_^2^* = 0.41; Attentiveness, Linear: η*_*p*_^2^* = 0.77; Quadratic: η*_*p*_^2^* = 0.17; 5th Order: η*_*p*_^2^* = 0.16). Positive mood is higher soon after waking, and declines with time awake.

For each positive mood, the effects of circadian displacement and when in that day testing took place interacted significantly (see Table 4 and Figure 7). The combined effects of both main effects on General Positive Affect and Attentiveness show very strong Quadratic-Linear trends for displacement and time of day respectively (Quadratic-Linear: η*_*p*_^2^* = 0.53, Quadratic-Linear: η*_*p*_^2^* = 0.57), with Cubic-Quadratic trends being the next largest effects sizes (General Positive Affect, Cubic-Quadratic: η*_*p*_^2^* = 0.39; Attentiveness, Cubic-Quadratic: η*_*p*_^2^* = 0.46). The Assuredness interaction reflects combinations of both 4th Order – Cubic trends (η*_*p*_^2^* = 0.46), or Linear-Linear (η*_*p*_^2^* = 0.34). Joviality is subject to Quadratic-Linear effects of displacement and test-point (η*_*p*_^2^* = 0.36; 4th Order-Cubic, η*_*p*_^2^* = 0.25). As is obvious from Figure 7, the effects of being awake on each positive mood intensify when the person sleep further away from their natural sleep time.

### Summary

**FIGURE 9:**
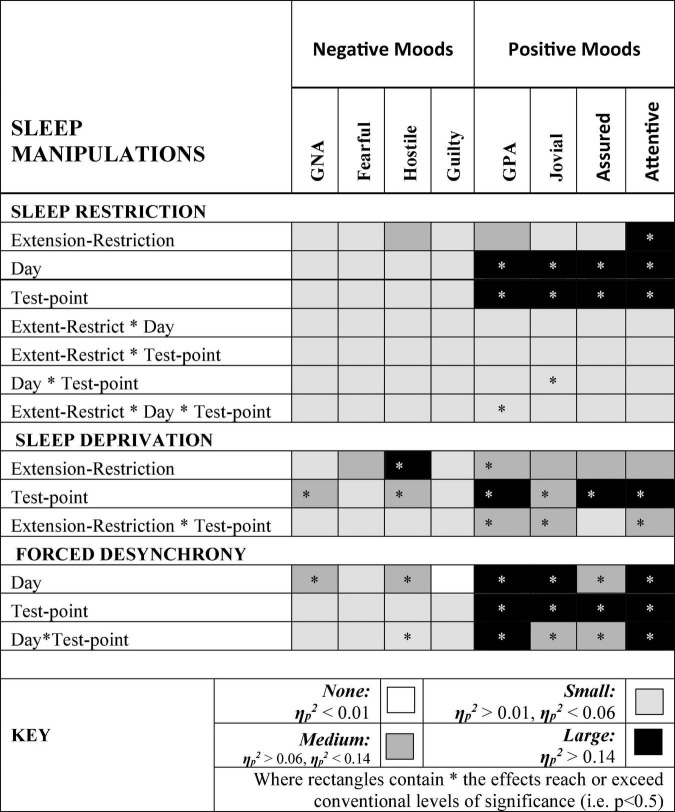
Effect sizes of sleep manipulations on Negative and Positive moods. Where rectangles contain * the effects reach or exceed conventional levels of significance (i.e. *p* < 0.5).

Figure 9 summarizes the effects on mood of the three paradigmatic sleep manipulations reported above, in terms of the relative scale of the effects sizes of each manipulation. There were small, but not necessarily significant, effects of each manipulation on each mood. The effects on positive moods were medium or large.

Hostility increased when sleep was restricted, when individuals were continuously awake for long periods having been sleep restricted, and when sleep was displaced from its typical timing. General Negative Affect and Hostility was unaffected by sleep restriction, but increased when time awake increased during sleep deprivation, when sleep was displaced from its typical timing. This suggests that the influence of circadian phase on General Negative Affect is particularly strong. Positive moods, particularly Attentiveness and General Positive Affect were reduced by sleep restriction, and when people were awake continuously there were large/medium effect of having been sleep restricted the previous week. In the absence of substantial sleep loss, but when the sleep opportunities available were out of phase with typical sleep, each of the four positive moods deteriorated substantially, with large/medium effects.

## Discussion

The re-analyzes reported above sought to address three issues that arise from previous literature: (a) previous studies provide little information about the specificity of any sleep manipulations with respect to particular positive mood states, beyond “vigor/energy” or “alertness”; (b) the effects on negative moods are inconsistent, but typically show an increase in Confusion and Fatigue when there are any at all, and (c) many previous results might reflect effects of circadian phase, rather than sleep loss.

The studies above show that different aspects of positive mood are more or less affected by shortening, preventing and displacing sleep. General Positive Affect and Attentiveness, the latter perhaps an echo of the declines in energy/vigor reported by others (e.g., Dinges et al., 1997), are affected by each manipulation. Joviality, on the other hand, seems more prone to circadian disruption, which is also an inevitable confound of most sleep deprivation studies. Assuredness is perhaps the least affected by our sleep manipulations.

Contrary to our previous reports, negative affect is influenced by sleep manipulations, but only particular aspects of negative affect- Hostility and General Negative Affect. PANAS adjectives do not easily translate to “Confusion,” but both Hostility and General Negative Affect increase as fatigue is operationally manipulated. Of the other two negative moods studied, Fear increased when poorly slept individuals attempt to remain awake for extended periods, while Guilt is influenced by all manipulations, but only to a small extent. It is worth noting that negative affect is generally quite low, and while it does increase for some negative moods and not others, it is possible that these idiosyncratic patterns of change, when combined into the Negative Affect measure obscures change in negative mood, as we and others have reported (e.g., Boivin et al., 1997; Lo et al., 2012; Santhi et al., 2016).

Manipulating circadian phase, without substantial sleep loss, shows extensive large effects on positive mood, General Positive Affect, Attentiveness, but also Joviality, Assuredness. General Negative Affect and Hostility also show medium sized effects of forced desynchrony. This substantial influence of circadian disruption is hardly surprising, but we believe the results above provide the clearest demonstration of this reported anywhere.

While we believe the sleep manipulations reported about are very robust, the measurement of mood states was, as is typical in the literature, a compromise. The short-form PANAS used here is typically used to measure the more global Positive and Negative Affect. Here, as described above, we re-categorized these into the more specific moods measured by the much longer PANAS-X, and this might have reduced our sensitivity to more specific moods than the full PANAS-X measure would allow. However, the internal reliabilities are excellent for each newly-created subscale, and what was measured does seem differentially sensitive to different sleep manipulations. Replicating these effects with the longer version of PANAS would be very useful. What we would caution against is the use of unvalidated single scales purporting to measure a specific mood. Inevitably, verbal items are interpreted in the context in which they occur, and may easily be suffused with irrelevant aspects of mood when these are not given the opportunity for expression. Studies that can only show that people express a lack of energy or vigor when we have deprived them of sleep can reveal very little about the subtleties of mood change as a function of sleep loss. We also question the usefulness of POMS in research contexts such as these, since it has little or no specificity with regard to positive moods.

We feel the re-analyzes reported above contribute substantial new insights to the literature: the relative insensitivity to chronic compared with acute sleep challenges; the very evident effects on mood of circadian phase without a substantial loss of sleep; the differential sensitivity of particular positive and negative moods to these challenges. However, we also need to emphasize that these effects are present in the relatively benign and highly controlled circumstances of the sleep laboratory, where there is no real emotional challenge- in contrast to what may be true of our everyday lives. The recent reports by Wong et al. (2021) and Shen et al. (2022), where moods are assessed repeatedly across weeks, provide a far more relevant insight into daily life. That acknowledged, without knowing what emotional challenges these individuals actually faced from day to day, it is difficult to be sure quite how helpful sleep might be in helping us to regulate our moods. What is clear from the present data is that even which consistently shortened sleep, people do not become more negative, and only do so when substantial sleep debt has been accumulated. People do, much more readily, become less positive in outlook. The data reported above also suggest that researchers need to be cautious when interpreting effects of time or day, or time awake, on mood- there are profound effects of circadian phase. Circadian markers would be an important addition to the very exciting ecological momentary analytic methods adopted in both studies, as would a more detailed investigation of the effects of menstrual phase. Several studies have reported sex differences in the effects of sleep-wake and circadian manipulations on measures of performance (e.g., Boivin et al., 2016; Santhi et al., 2016; Vidafar et al., 2018). In the present analyzes we have not explored these effects because, in contrast to the aforementioned studies, the current analyzes are based on only one assessment tool (i.e., PANAS). This and the relatively small number of men and women precludes a robust assessment of sex differences.

While the studies reported above both included similar numbers of people self-identifying as men or women, and carried out pregnancy tests to avoid unnecessary risk to participants or potential progeny, neither study was statistically powered to assess sex differences, nor was there any requirement on participants to report in which part of their menstrual cycle they were being tested. Far from ignoring the importance of possible sex differences on affect and mood, we believe without robust measures of hormonal changes typical of menstruating women, any serendipitous findings with regard to mood and sex are likely to understate, confuse or mislead.

Finally, the differentiation within and between positive and negative moods reported above has implications for theoretical accounts of affect and mood which rely on the orthogonality of both. Manipulations of sleep and circadian phase clearly demonstrate their independence, but the PANAS framework theorizes that low arousal can be reflected in lower levels of Positive or Negative Affect, the data reported above render this claim empirically questionable, adding to its conceptual confusion.

## Conclusion

Reductions in nightly sleep duration, extended sleep deprivation and sleeping out of phase with one’s normal sleep-wake routine, all influence mood in general, and particular mood states. Theoretical conceptualizations of mood which differentiate between an energetic and a more valence pleasant/positive state (or its opposite), are supported by the studies reported above, but when at least one of these dimensions is confounded with an essential and highly variable aspect of everyday life-sleep. Such theorizing on the structure of mood might benefit from considering what happens to mood when sleep is shifted, shortened, or removed.

## Data Availability Statement

The original contributions presented in the study are included in the article/supplementary material, further inquiries can be directed to the corresponding author.

## Ethics Statement

The studies involving human participants were reviewed and approved by University of Surrey Research Ethics Committee. The patients/participants provided their written informed consent to participate in this study.

## Author Contributions

D-JD and JG respectively were principal and co-investigators on the original studies, designed and oversaw the protocol, data collection, statistical analyzes, and reporting. JL, NS, and ASL collected most of the data reported. JG conceived the current study and carried out the statistical analyzes. All authors contributed to the article and approved the submitted version.

## Conflict of Interest

The authors declare that the research was conducted in the absence of any commercial or financial relationships that could be construed as a potential conflict of interest.

## Publisher’s Note

All claims expressed in this article are solely those of the authors and do not necessarily represent those of their affiliated organizations, or those of the publisher, the editors and the reviewers. Any product that may be evaluated in this article, or claim that may be made by its manufacturer, is not guaranteed or endorsed by the publisher.

## Footnotes

1 The original PANAS (Positive and Negative Affect Schedule), was a formulation of 20 items (Watson et al., 1988) measuring but was later extended to 60 items (PANAS-X) allowing the measurement of Fear, Sadness, Guilt, Hostility (i.e., four Basic Negative Emotions), Joviality, Self-Assurance, Attentiveness (i.e., three Basic Positive Emotion scales) as well as four “Other Affective Scales”: Shyness, Fatigue, Surprise, and Serenity. POMS (Profile of Mood States, McNair et al., 1971), comprises 65 items and yields a total mood index, together with a single index of positive mood (Vigor/Activity) and five indices of negative mood (Tension/Anxiety, Depression/Dejection, Anger/Hostility, Fatigue/Inertia, and Confusion/Bewilderment).

## References

[B1] Akerstedt T. Axelsson J. Lekander M. Orsini N. Kecklund G. (2013). The daily variation in sleepiness and its relation to the preceding sleep episode–a prospective study across 42 days of normal living. *J. Sleep Res.* 22 258–265. 10.1111/jsr.12014 23205895

[B2] American Psychiatric Association (2013). *Diagnostic and Statistical Manual of Mental Disorders 5 th Edn.* Arlington, VA: American Psychiatric Publishing.

[B3] Barrett L. F. Russell J. A. (1999). The structure of current affect: controversies and emerging consensus. *Curr. Dir. Psychol. Sci.* 8 10–14. 10.1088/1478-3975/10/4/040301

[B4] Boivin D. B. Czeisler C. A. Dijk D. J. Duffy J. F. Folkard S. Minors D. S. (1997). Complex interaction of the sleep-wake cycle and circadian phase modulates mood in healthy subjects. *Arch. Gen. Psychiatry* 54 145–152. 10.1001/archpsyc.1997.01830140055010 9040282

[B5] Boivin D. B. Shechter A. Boudreau P. Begum E. A. Ng Ying-Kin N. M. (2016). Diurnal and circadian variation of sleep and alertness in men vs. naturally cycling women. *Proc. Natl. Acad. Sci.* 113 10980–10985. 10.1073/pnas.1524484113 27621470 PMC5047150

[B6] Cohen J. (1988). *Statistical Power Analysis for the Behavioral Sciences.* New York, NY: Routledge Academic.

[B7] Duffy J. F. Dijk D. J. (2002). Getting through to circadian oscillators: why use constant routines? *J. Biol. Rhythms* 17 4–13. 10.1177/074873002129002294 11837947

[B8] Dijk D. J. Groeger J. A. Stanley N. Deacon S. (2010). Age-related reduction in daytime sleep propensity and nocturnal slow wave sleep: Implications for insomnia. *Sleep* 33 211–223. 10.1093/sleep/33.2.211 20175405 PMC2817908

[B9] Dinges D. F. Pack F. Williams K. Gillen K. A. Powell J. W. Ott G. E. (1997). Cumulative sleepiness, mood disturbance, and psychomotor vigilance performance decrements during a week of sleep restricted to 4-5 hours per night. *Sleep.* 20 267–277. 9231952

[B10] Groeger J. A. Stanley N. Deacon S. Dijk D. J. (2014). Dissociating effects of global SWS disruption and healthy aging on waking performance and daytime sleepiness. *Sleep* 37 1127–1142. 10.5665/sleep.3776 24882908 PMC4015387

[B11] Groeger J. A. Viola A. U. Lo J. C. Y. Archer S. N. von Schantz M. Dijk D. J. (2008). Early morning executive functioning during sleep deprivation is compromised by a PERIOD3 polymorphism. *Sleep* 31 1159–1167. 18714788 PMC2542962

[B12] Harous C. Roach G. D. Kontou T. G. Montero A. J. Stuart N. Sargent C. (2021). Consecutive nights of moderate sleep loss does not affect mood in healthy young males. *Clocks Sleep* 3 442–448. 10.3390/clockssleep3030031 34449566 PMC8395486

[B13] Hertenstein E. Feige B. Gmeiner T. Kienzler C. Spiegelhalder K. Johann A. (2019). Insomnia as a predictor of mental disorders: a systematic review and meta-analysis. *Sleep Med. Rev.* 43 96–105. 10.1016/j.smrv.2018.10.006 30537570

[B14] IBM Corp. (2021). *IBM SPSS Statistics for Windows, Version 28.0.0.* Armonk, NY: IBM Corp.

[B15] Kaida K. Niki K. (2014). Total sleep deprivation decreases flow experience and mood status. *Neuropsychiatr. Dis. Treat.* 10 19–25. 10.2147/NDT.S53633 24376356 PMC3865143

[B16] Kaufmann C. Agalawatta N. Bell E. Malhi G. S. (2020). Getting emotional about affect and mood. *Aust. N. Z. J. Psychiatry* 54 850–852. 32735173 10.1177/0004867420943943

[B17] Larsen R. J. Diener E. (1992). “Promises and problems with the circumplex model of emotion,” in *Review of personality and social psychology: emotion*, ed. Clark M. S. (Newbury Park, CA: SAGE), 25–59. 10.1177/0004867420943943

[B18] Li B. Z. Cao Y. Zhang Y. Chen Y. Gao Y. H. Peng J. X. (2021). Relation of decreased functional connectivity between left thalamus and left inferior frontal gyrus to emotion changes following acute sleep deprivation. *Front. Neurol.* 12:642411. 10.3389/fneur.2021.642411 33716944 PMC7952868

[B19] Lo C. Y. Groeger J. A. Santhi N. Arbon E. L. Lazar A. S. Hasan S. (2012). Effects of partial and acute total sleep deprivation on performance across cognitive domains, individuals and circadian phase. *PLoS One* 7:e45987. 10.1371/journal.pone.0045987 23029352 PMC3454374

[B20] McNair D. M. Lorr M. Droppelman L. F. (1971). *Manual for the Profile of Mood States.* San Diego, CA: Educational and Industrial Testing Service.

[B21] Meney I. Waterhouse J. Atkinson G. Reilly T. Davenne D. (1988). The effect of one night’s sleep deprivation on temperature, mood, and physical performance in subjects with different amounts of habitual physical activity. *Chronobiol. Int.* 15 349–363. 10.3109/07420529808998695 9706412

[B22] Musiek E. S. Holtzman D. M. (2016). Mechanisms linking circadian clocks, sleep, and neurodegeneration. *Science.* 354 1004–1008. 10.1126/science.aah4968 27885006 PMC5219881

[B24] Russell J. A. (1980). A circumplex model of affect. *J. Pers. Soc. Psychol.* 39 1161–1178. 10.1037/h0077714

[B25] Saksvik-Lehouillier I. Saksvik S. B. Dahlberg J. Tanum T. K. Ringen H. Karlsen H. R. (2020). Mild to moderate partial sleep deprivation is associated with increased impulsivity and decreased positive affect in young adults. *Sleep* 43:zsaa078. 10.1093/sleep/zsaa078 32306048 PMC7551297

[B26] Santhi N. Lazar A. S. McCabe P. J. Lo J. C. Groeger J. A. Dijk D. J. (2016). Sex differences in the circadian regulation of sleep and waking cognition in humans. *PNAS* 113 E2730–E2739. 10.1073/pnas.1521637113 27091961 PMC4868418

[B27] Shen L. Wiley J. F. Bei B. (2022). Sleep and affect in adolescents: bidirectional daily associations over 28-day ecological momentary assessment. *J Sleep Res.* 31:e13491. 10.1111/jsr.13491 34585468

[B28] Thayer R. E. (1989). *The Biopsychology of Mood and Activation.* New York, NY: Oxford University Press.

[B29] Vandewalle G. Archer S. N. Wuillaume C. Balteau E. Degueldre C. Luxen A. (2009). Functional magnetic resonance imaging-assessed brain responses during an executive task depend on interaction of sleep homeostasis, circadian phase, and PER3 genotype. *J. Neurosci.* 29 7948–7956. 10.1523/JNEUROSCI.0229-09.2009 19553435 PMC6666044

[B30] Vidafar P. Gooley J. J. Burns A. C. Rajaratnam S. M. W. Rueger M. Van Reen E. (2018). Increased vulnerability to attentional failure during acute sleep deprivation in women depends on menstrual phase. *Sleep* 41:zsy098. 10.1093/sleep/zsy098 29790961 PMC6093460

[B32] Watson D. Tellegen A. (1985). Toward a consensual structure of mood. *Psychol. Bull.* 98 219–235. 10.1037//0033-2909.98.2.219 3901060

[B33] Watson D. Clark L. A. Tellegen A. (1988). Development and validation of brief measures of positive and negative affect: The PANAS scales. *J. Pers. Soc. Psychol.* 54 1063–1070. 10.1037/0022-3514.54.6.1063 3397865

[B34] Wong P. M. Hasler B. P. Kamarck T. W. Wright A. G. C. Hall M. Carskadon M. A. (2021). Day-to-day associations between sleep characteristics and affect in community dwelling adults. *J. Sleep Res.* 30:e13297. 10.1111/jsr.13297 33588521 PMC8637582

[B35] Wundt W. (1912/1924). *An Introduction to Psychology (R. Pintner, Trans.).* London: Allen and Unwin. 10.1037/13784-000

